# Retraction Note: Inhibitors of apoptosis proteins in experimental benign prostatic hyperplasia: effects of *Serenoa repens*, selenium and lycopene

**DOI:** 10.1186/s12929-024-01066-x

**Published:** 2024-08-02

**Authors:** Letteria Minutoli, Domenica Altavilla, Herbert Marini, Mariagrazia Rinaldi, Natasha Irrera, Gabriele Pizzino, Alessandra Bitto, Salvatore Arena, Sebastiano Cimino, Francesco Squadrito, Giorgio Ivan Russo, Giuseppe Morgia

**Affiliations:** 1 Department of Clinical and Experimental Medicine, University of Messina, 98125 Messina, Italy; 2 Department of Paediatric, Gynaecological, Microbiological and Biomedical Sciences, University of Messina, 98125 Messina, Italy; 3 Department of Urology, Polyclinic Hospital, University of Catania, 95100 Catania, Italy

**Retraction Note: Journal of Biomedical Science (2014) 21:19** **http://www.jbiomedsci.com/content/21/1/19**

The Editor-in-Chief retracted this article because of concerns regarding a number of figures presented in this work. These concerns call into question the article's overall scientific soundness. An investigation conducted after its publication discovered similarities in the six gels presented in Figure 1. The Editors-in-Chief therefore no longer have confidence in the integrity of the research presented in this article.

Giorgio Ivan Russo agrees to this retraction. Alessandra Bitto and Giuseppe Morgia disagree to this retraction. Letteria Minutoli, Herbert Marini, Natasha Irrera, Salvatore Arena, and Sebastiano Cimino have not replied to correspondence from the Publisher. The Publisher was unable to contact Mariagrazia Rinaldi and Gabriele Pizzino. Alessandra Bitto has also informed the Publisher that Domenica Altavilla and Francesco Squadrito have passed away.

